# Periodic Fluctuations in the Incidence of Gastrointestinal Cancer

**DOI:** 10.3389/fonc.2021.558040

**Published:** 2021-03-23

**Authors:** Mun-Gan Rhyu, Jung-Hwan Oh, Tae Ho Kim, Joon-Sung Kim, Young A Rhyu, Seung-Jin Hong

**Affiliations:** ^1^ Department of Microbiology, College of Medicine, The Catholic University of Korea, Seoul, South Korea; ^2^ Department of Internal Medicine, College of Medicine, The Catholic University of Korea, Seoul, South Korea; ^3^ Department of Internal Medicine, Konkuk University School of Medicine, Seoul, South Korea

**Keywords:** gastric cancer, colon cancer, mass screening, *Helicobacter pylori*, adult stem cells

## Abstract

**Purpose:**

Native stem cells can be periodically replaced during short and long epigenetic intervals. Cancer-prone new stem cells might bring about periodic (non-stochastic) carcinogenic events rather than stochastic events. We investigated the epigenetic non-stochastic carcinogenesis by analyzing regular fluctuations in lifelong cancer incidence.

**Materials and Methods:**

Korean National Cancer Screening Program data were collected between 2009 and 2016. Non-linear and log-linear regression models were applied to comparatively evaluate non-stochastic and stochastic increases in cancer incidence. Prediction performances of regression models were measured by calculating the coefficient of determination, R^2^.

**Results:**

The incidence of gastric and colorectal cancers fluctuated regularly during both short (8 years) and long (20 years) intervals in the non-linear regression model and increased stochastically in the log-linear regression model. In comparison between the 20-year interval fluctuation model and the stochastic model, R^2^ values were higher in the 20-year interval fluctuation model of men with gastric cancer (0.975 vs. 0.956), and in the stochastic model of men with colorectal cancer (0.862 vs. 0.877) and women with gastric cancer (0.837 vs. 0.890) and colorectal cancer (0.773 vs. 0.809). Men with gastric cancer showed a high R^2^ value (0.973) in the 8-year interval fluctuation model as well.

**Conclusion:**

Lifelong incidence of gastrointestinal cancer tended to fluctuate during short and long intervals, especially in men with gastric cancer, suggesting the influence of an epigenetic schedule.

## Introduction

Cancer is thought to arise *via* a multistage process involving the sequential accumulation of random carcinogenic events (1, 2). Age-specific cancer incidence increases exponentially after 40 years (3). A stochastic multistage model has proposed that a set of random carcinogenic events triggers an exponential increase in the incidence of cancer (4). The adenoma-adenocarcinoma sequence is a well-known example of multistage gastrointestinal carcinogenesis (5, 6). It is noteworthy that early-onset gastrointestinal cancers commonly arise without precancerous lesions (7, 8). Of the two distinct types of gastric cancer, diffuse and intestinal, diffuse-type cancer is associated with an early onset of cancer without accompanying precancerous lesions (9). Adenomatous lesions are less common in colorectal cancer patients younger than 55 years (7). The so-called *de novo* carcinogenesis appears to occur with no precursors in middle age when multistage, slow developing carcinogenesis is not yet valid. Consequently, lifelong carcinogenesis begins to accelerate after the age of 40, and *de novo* carcinogenesis is prominent during the early period of accelerated carcinogenesis.

As a part of aging process, native stem cells in reducing their self-renewal capacity are replaced with new stem cells (10). The newly fixed stem cells are phenotypically unstable and prone to transform into cancer stem cells (10), which are stabilized by cell division-dependent methylation (11). *Helicobacter pylori*-associated methylation of CpG-island genes increases from 40 years of age (12) even though *H. pylori*-infection takes place in young adults (13). The methylation changes occur extensively over the antrum and body of the stomach (14). This suggests that gastric mucosal stem cells are epigenetically programmed to be replaced with new stem cells in a particular age. An epigenetic schedule initiating after the age of 40 seems to generate unstable stem cells associated with *de novo* carcinogenesis in the early period and subsequently stabilized stem cells associated with multistage carcinogenesis.

Previous mathematical analyses on age-specific incidences of cancer have classified the accelerated carcinogenesis into the first exponential phase and the second non-exponential phase (15, 16). Unstable new stem cells in an early period may be responsible for the early exponential phase of accelerated carcinogenesis, and subsequently stabilized at non-exponential phase. A previous methylation study demonstrated that stem cells in the colonic glandular structures are replaced every 8 years (17). This indicates another epigenetic schedule with short-term cycles in addition to the two long-term phases. The long- and short-term epigenetic schedules can bring about exponential and non-exponential phases periodically during lifelong carcinogenesis. However, few population-based studies have noted periodic fluctuations in age-specific cancer incidence. A small proportion of unstable stem cells in the early period of the epigenetic cycle might result in ambiguous cycles of exponential and non-exponential phases. DNA methylation of CpG island genes significantly increases in the gastric mucosa infected with *H. pylori*. A high number of new unstable stem cells seem to participate in *H. pylori*-associated gastric carcinogenesis, which is expected to produce visible epigenetic cycles.

In Korea, *H. pylori*-infection rate and gastric cancer incidence are high (18). Gastric cancer data of Korea may be useful to demonstrate regular fluctuations in cancer incidence. The Korean National Cancer Screening Program (KNCSP) conducts biennial gastric cancer screening as well as annual colorectal cancer screening (19, 20). We employed non-linear regression and log-linear regression to identify non-stochastic fluctuations and stochastic exponential growth in cancer incidence, respectively (4). Putative fluctuations in gastric and colorectal cancer incidences were evaluated by non-linear models at long (20 years) and short (8 years) intervals. Prediction performances of non-stochastic and stochastic models were comparatively assessed according to cancer types and sex.

## Materials and Methods

### Cancer Screening Data

Gastric and colorectal cancer screening data were obtained from the KNCSP. The gastric cancer screening program, which was launched in 1999, recommends biennial screening *via* gastric endoscopy or upper gastrointestinal series for adults aged ≥40 years (19). Colorectal cancer screening, which was initiated in 2004, recommends annual fecal occult blood testing for adults aged ≥50 years and administers colonoscopies or double contrast barium enemas in patients with positive fecal occult blood tests (20). The KNCSP’s diagnostic criteria for gastrointestinal cancer were revised in 2009 to allow the consideration of pathologic findings. Accordingly, the KNCSP data of patients registered between 2009 and 2016 were included in this study; patients previously diagnosed with gastric or colorectal cancers were excluded. Patients were determined to have a synchronous gastric and colorectal cancer when both were detected simultaneously during screening.

### Non-linear and Log-Linear Cancer Incidence Models

Periodic fluctuations in the age-specific numbers of patients diagnosed with gastric or colorectal cancers were modeled using the non-linear regression where the age-specific incidence curve *λ*(age) is a combination of periodic fluctuation function (*f*_p_) and age-related stem-cell enrichment function (*f*_s_):

$$\lambda \left(age\right)=f_{p}\left(age\right)+\;f_{s}\left(age\right)=\beta_{1}cos(\;\frac{2\pi }{\theta }X_{age})+\beta_{2}X_{age}+\;\gamma +\epsilon ,$$

in which *λ* corresponds to the incidence rate as a function of age, functions *f*_p_ and *f*_s_ are in terms of the year of incidence, *θ* is the year of stem cell replacement, *β*_1_, *β*_2_, and γ are unknown parameters, and ϵ is error. The function *f*_p_ represents the periodic cycle according to the period of stem cell replacement, and the function *f*_s_ represents the increasing number of new stem cells depending on age. The parameter was calculated using non-linear regression using the Gauss-Newton method.

Stochastic model of random occurrences in the cancer stem cells was evaluated using log-linear regression as follows:

$$Log\lambda \left(age\right)=\beta Log(X_{age})+\;\gamma \;+\;\epsilon ,$$

in which *λ* corresponds to the incidence rate as a function of age, *β* and γ are unknown parameters, and ϵ is error (4). Approximate significance levels for non-linear and log-linear regression models were calculated using the F distribution, and *P* < 0.05 was considered significant. R-squared statistics were computed to assess the goodness-of-fit of regression models. Mean squared errors were calculated to estimate the error variances of regression models.

### Growth Rate of Cancer Incidence

To determine the actual fluctuation curves inherent to the age-dependent increases in cancer diagnoses, the growth rate (%) of the age-specific cancer incidence was calculated using the following formula: (incidence rate of the target age group – incidence rate of the previous age group)/the incidence rate of previous age group × 100.

### Ethics Statement

Our investigation of the cancer screening data was reviewed and approved by the Institutional Review Board of The Catholic University of Korea, Songeui Campus (approval No. MC18EESI0059).

## Results

### Incidence of Gastrointestinal Cancers

A total of 44,998,900 men and 46,838,806 women aged ≥40 years were invited to participate in the KNCSP for gastric cancer between 2009 and 2016 (**Table S1**). The incidence rate of new gastric cancer diagnoses per 100,000 participants was 2.5-fold higher in men (210) than in women (83). Furthermore, the KNCSP for colorectal cancer invited 44,038,275 men and 47,974,535 women aged ≥50 years between 2009 and 2016 (**Table S2**). The colorectal cancer incidence rate was 2.3-fold higher in men (84) than in women (37).

**Figure 1:**
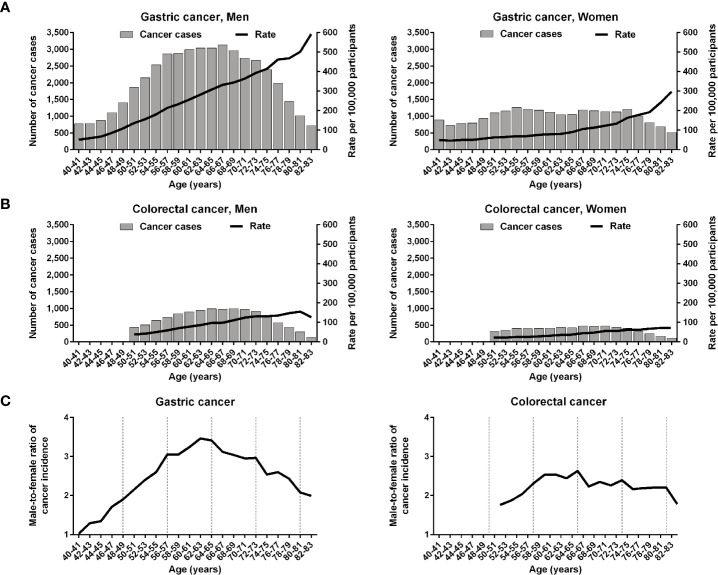
Age-specific incidences of gastric and colorectal cancers in the Korean National Cancer Screening Program between 2009 and 2016. **(A, B)** The incidences of gastric cancer and those of colorectal cancer are depicted at 2-year intervals. **(C)** Male-to-female ratios of gastric and colorectal cancer incidences are plotted. The vertical dashed lines indicate 8-year intervals after 40 years of age.

The age-specific incidence rates at 2-year age intervals were plotted using the biennial gastric cancer and the annual colorectal cancer screening data. The incidence curves of both gastric and colorectal cancers continuously grew in an age-dependent manner (**Figures 1A, B**). The male-to-female ratio of cancer incidence rates increased steadily to peak at similar ages for gastric cancer (62–63 years) and colorectal cancer (64–65 years) (**Figure 1C**). Age-dependent increases in cancer incidences were rapid before 64 years of age in men and after 64 years of age in women; accordingly, we categorized the participants into 2 age groups (<64 and ≥64 years) to address the two distinct phases of cancer incidences.

### Regression Models of Age-Specific Cancer Incidence

**Figure 2:**
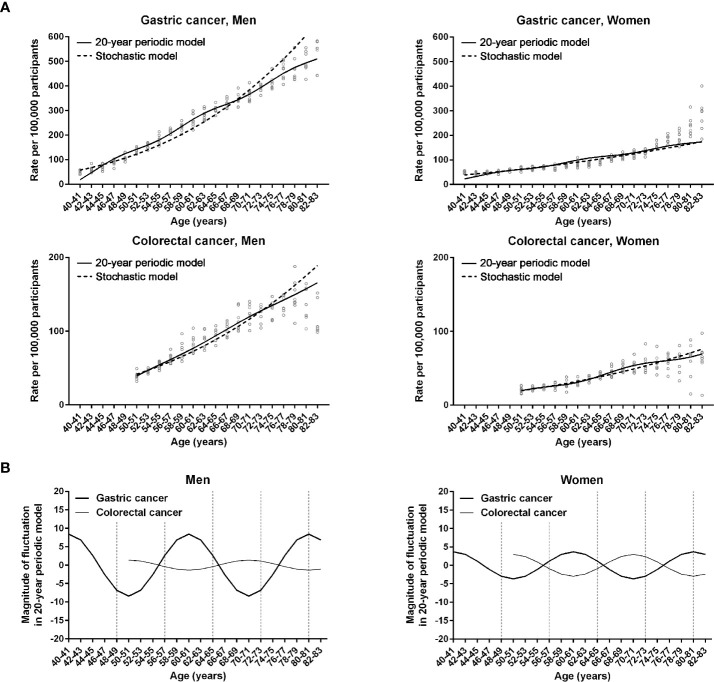
Non-linear regression models for 20-year fluctuation intervals in gastric and colorectal cancers. **(A)** Comparison of non-linear periodic model (solid lines) and log-linear stochastic model (dashed lines). Dots represent observed incidence rates of gastric and colorectal cancers in the Korean National Cancer Screening Program between 2009 and 2016. **(B)** Fluctuation magnitudes of periodic fluctuation function in models 20-year-interval regression equation. Vertical dashed lines indicate 8-year intervals after 40 years of age.

**Figure 3:**
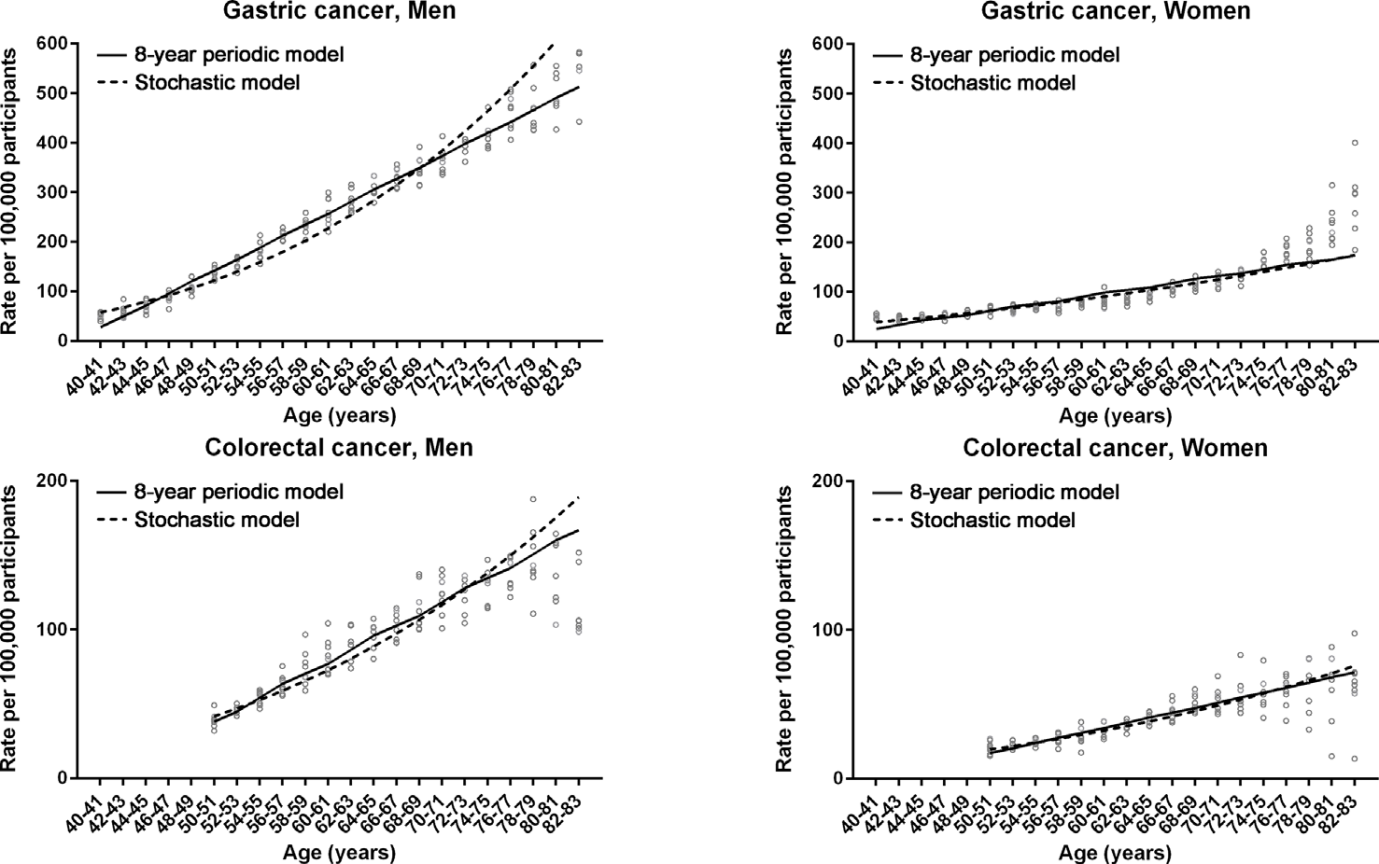
Non-linear regression models for 8-year fluctuation intervals in gastric and colorectal cancers. Non-linear periodic model (solid lines) and log-linear stochastic model (dashed lines) are compared. Dots represent observed incidence rates of gastric and colorectal cancers in the Korean National Cancer Screening Program between 2009 and 2016.

The non-linear regression was designed for periodic (non-stochastic) incidence models of long (20 years) and short (8 years) intervals (**Table 1**). Long- and short-interval fluctuations in age-specific incidences were regular for gastric and colorectal cancers in both men and women, with statistical significance (*P* < 0.0001, R^2^ > 0.7) (**Figures 2A**, **3**). When the stochastic cancer occurrence was evaluated using log-linear regression, gastric and colorectal cancers were found to exponential increase, with statistical significance (*P* < 0.0001 and R^2^ > 0.8) (**Figures 2A**, **3**; **Table 1**). The R^2^ value of men with gastric cancer was higher in long- (0.975) and short-interval (0.973) non-stochastic models than in the stochastic model (0.956). The stochastic model revealed higher R^2^ values in women with gastric cancer (0.890 vs. 0.837 and 0.834), and both men (0.877 vs. 0.862 and 0.862) and women (0.809 vs. 0.773 and 0.756) with colorectal cancer than the non-stochastic models for long and short intervals.

**Table 1:** Equations of non-linear and log-linear regression models for the gastric and colorectal cancers in Korean National Cancer Screening Program 2009–2016.

Cancer type/Sex	Equation	R^2^	*P*	Error Rate (%)	Mean squared error
**20-year interval fluctuation model**					
Gastric cancer/Men	$Y\;=\;8.4Cos\left(0.1\pi X_{age}\right)\;+\;11.6X_{age}-\;439.3$	0.975	<0.001	2.5	463.0
Gastric cancer/Women	$Y\;=\;3.7Cos\left(0.1\pi X_{age}\right)\;+\;3.5X_{age}-\;116.4$	0.837	<0.001	16.3	328.4
Colorectal cancer/Men	$Y\;=\;-1.3Cos\left(0.1\pi X_{age}\right)\;+\;4.0X_{age}-\;163.7$	0.862	<0.001	13.8	197.2
Colorectal cancer/Women	$Y\;=\;-\;3.0Cos\left(0.1\pi X_{age}\right)\;+\;1.7X_{age}-\;69.4$	0.773	<0.001	22.7	66.1
**8-year interval fluctuation model**					
Gastric cancer/Men	$Y=\;1.3Cos\left(0.25\pi X_{age}\right)\;+\;11.6X_{age}-\;436.2$	0.973	<0.001	2.7	497.7
Gastric cancer/Women	$Y\;=\;-1.8Cos\left(0.25\pi X_{age}\right)\;+\;3.5X_{age}-\;113.9$	0.834	<0.001	16.6	333.5
Colorectal cancer/Men	$Y\;=\;1.4Cos\left(0.25\pi X_{age}\right)\;+\;4.0X_{age}-\;162.9$	0.862	<0.001	13.8	197.3
Colorectal cancer/Women	$Y\;=\;0.2Cos\left(0.25\pi X_{age}\right)\;+\;1.7X_{age}-\;67.6$	0.758	<0.001	24.2	70.4
**Log linear model**					
Gastric cancer/Men	$Log\left(Y\right)\;=\;3.4Log\left(X_{age}\right)\;-\;8.5$	0.956	<0.001	4.4	0.021
Gastric cancer/Women	$Log\left(Y\right)\;=\;2.1Log\left(X_{age}\right)\;-\;4.0$	0.890	<0.001	11.0	0.021
Colorectal cancer/Men	$Log\left(Y\right)\;=\;3.1Log\left(X_{age}\right)\;-\;8.2$	0.877	<0.001	12.3	0.024
Colorectal cancer/Women	$Log\left(Y\right)\;=\;2.7Log\left(X_{age}\right)\;-\;7.7$	0.809	<0.001	19.1	0.033

The magnitudes of the fluctuation curves in the cancer incidence models were evaluated by calculating the 20-year fluctuation functions of the non-linear regression equations. The directions of 20-year-interval cycles were different between the two cancer types in both men and women (**Figure 2B**).

### The Growth Rate of Age-Specific Cancer Incidence

**Figure 4:**
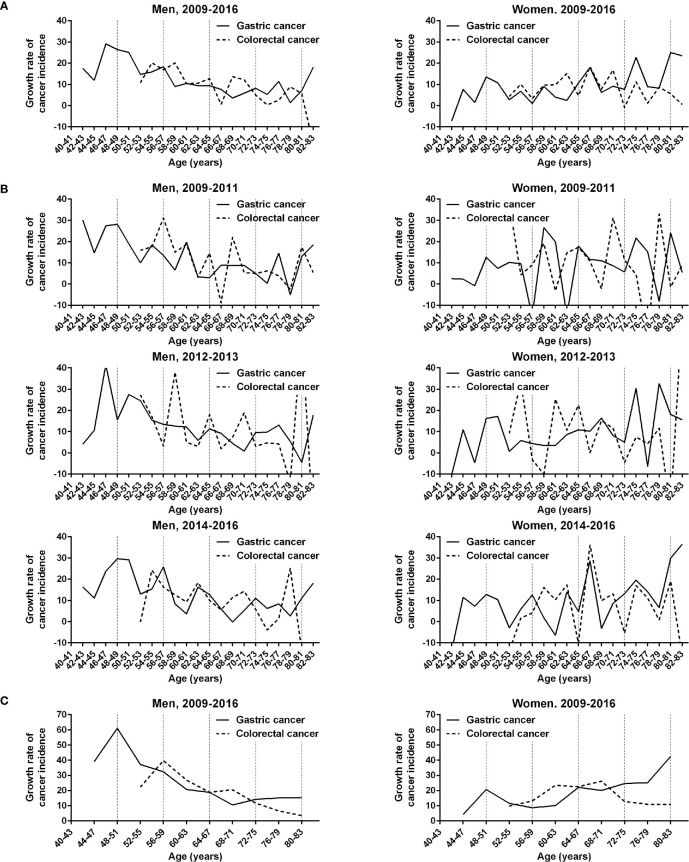
Comparison of the growth rates of gastric and colorectal cancer incidences in men and women in the Korean National Cancer Screening Program between 2009 and 2016. **(A)** Growth rates of gastric and colorectal cancer incidences are plotted at 2-year intervals. **(B)** Growth rate curves are plotted according to the Korean National Cancer Screening Program year subgroups 2009–2011, 2012–2013, and 2014–2016. **(C)** Growth rates of the 2 cancer incidences are plotted at 4-year intervals. Vertical dashed lines indicate 8-year intervals after 40 years of age.

The complex fluctuations inherent in age-dependent cancer incidences were reviewed relative to the growth rate of age-specific cancer incidence (**Figure 4A**). Overall, the periodicities in the growth rate curves of gastric and colorectal cancers were obscure in men and women. The growth rate curves of the two cancer types tended to reach its trough near the boundary (64 years) between the two distinct phases. The KNCSP-registered patients were divided into three subgroups based on the time of diagnosis: old (2009 to 2011), middle (2012 to 2013), and recent (2014 to 2016). The KNCSP data were compared among the 3 screening year subgroups (**Figure 4B**). The growth rate curve for gastric cancer was at its trough within an age range of 64 and 71 years among all three KNCSP year subgroups and that for colorectal cancer between the ages of 66 and 67 years. Consequently, the growth rate curves of the two cancer types demonstrated two distinct phases of similar boundaries.

The growth rate curves of gastric and colorectal cancer incidences tended to peak regularly at short-term intervals in the first phase in the recent screening years compared to the old screening years (**Figure 4B**). In addition, the peaks of the short-term fluctuations were found at similar ages on the growth rate curves of gastric cancer (56–57 and 62–63 years) and colorectal cancer (54–55 and 62–63 years). Notably, the growth rate curves in men were decoupled between the two cancer types at the first short-term fluctuation in the second phase (68–71 years). In an analysis of 4-year intervals, the growth rate curves of the two cancers were also found to be decoupled in the second phase (68–71 years) in men (**Figure 4C**).

### Synchronous Gastric and Colorectal Cancer

**Figure 5:**
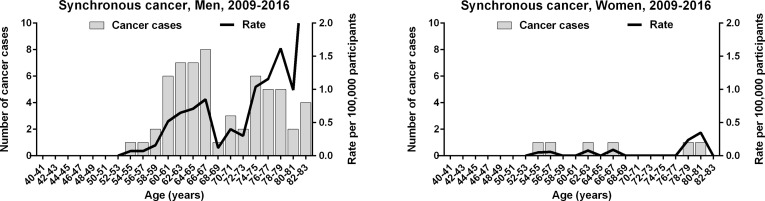
Age-specific incidence of synchronous gastric and colorectal cancer in the Korean National Cancer Screening Program between 2009 and 2016.

We investigated a tendency toward the simultaneous cancerization of gastric and colorectal cancers. Between 2009 and 2016, a total of 66 individuals were found to have a synchronous gastric cancer and colorectal cancer, which corresponded to 0.11% and 0.36% of patients with gastric cancer and colorectal cancer, respectively. As colorectal cancer screening is conducted in individuals 50 years and older, the youngest patient with a synchronous cancer was 54 years of age. The number of men with a synchronous gastric and colorectal cancer was 10-fold higher than women with the same (60 vs. 6). Among men, there were a high number of synchronous cancers between the ages of 60 and 67 (28 patients) and 74 and 79 years (16 patients); a low number were between the ages of 68 and 73 years (6 patients) (**Figure 5**). As such, the age-specific incidence of synchronous cancer dropped within a limited age range shortly after the 64 year cut-off age.

## Discussion

In this population-based study, the age-specific incidence of gastric and colorectal cancers fluctuated regularly during long-term (20-years) and short-term (8-years) intervals. Non-linear regression models for non-stochastic carcinogenic events were more accurate during both the long- and short-term intervals in men with gastric cancer compared with log-linear regression models for stochastic carcinogenic events. An epigenetic program that induces regular replacement of stem cells appears to periodically accelerate carcinogenesis as a part of the aging process. It has been long believed that stochastic accumulation of genetic alterations triggers an age-dependent exponential increase in cancer diagnoses. Accordingly, log-linear regression models for stochastic events were more accurate in both men and women with colorectal cancers. Women with gastric cancer also showed a higher R^2^ value for stochastic events. This indicates that stochastic genetic alterations have widespread effects on gastrointestinal carcinogenesis. Therefore, it is likely that an epigenetic schedule exerts a strong influence particularly on men with gastric cancer.

*H*. *pylori*-associated methylation increases from 40 years of age and reaches a peak level at 60 years of age, serving as a marker for recruitment of new stem cells (12, 14). The stomach-specific gene *TFF3* was found to be periodically methylated among patients with mild (51 ± 9 years), moderate (58 ± 9 years), and severe (63 ± 10 years) levels of gastric mucosal atrophy despite non-periodic progression of mucosal atrophy (mild, 51 ± 9 years; moderate, 54 ± 9 years; and severe, 65 ± 10 years) (21). The periodic methylation of *TFF3* is consistent with the 8-year-interval fluctuations during the first long-term phase. The periodic replacement of stem cells may account for the short-term fluctuations in the development of gastrointestinal cancer.

Among the Korean population, the incidence of gastric cancer is the highest in men (22). The prevalence of *H. pylori* infection is high in Korea compared with western countries, despite reducing rates in recent decades (23). During the period of stem cell replacement under an epigenetic program, *H. pylori* infection may promote a replacement of cancer-prone new stem cells. High prevalence of *H. pylori* infection is thought to increase the prediction performance of the non-stochastic model in gastric cancer patients. Comparative analysis of cancer incidence fluctuations among countries based on the prevalence of *H. pylori* infection will be needed to verify the non-stochastic model for gastrointestinal cancer.

The incidences of gastric and colorectal cancers are approximately two times higher in men than in women (24). In particular, men and women exhibit similar seropositivity rates of *H. pylori*, the major cause of gastric cancer (25). Somatic mutation rates of adult stem cells are similar between men and women (26). Environmental and genetic factors seem to have weak influence on sex-specific development of gastrointestinal cancer. A previous genome-wide study shows that DNA methylation is denser in men than in women (27). Female X-chromosome inactivation may restrain the methylation of housekeeping genes in autosome. It is likely that epigenetic structures in men facilitate the stabilization of new stem cell phenotypes increasing the number of cancer-prone new stem cells during the period of stem cell replacement.

Bone-marrow stem cells of male donors are able to differentiate into mesenchymal cells and glandular cells in the gastrointestinal mucosa of female recipients (28). Marrow-derived stem cells with long-term renewal capacity may replace old mesenchymal stem cells over an extensive area of the gastrointestinal mucosa. In fact, *LGR5*, a potential stem cell marker, is commonly expressed in the gastric and colonic mucosa in addition to marrow stem cells (29, 30). New mesenchymal stem cells remain quiescent in a niche subjacent to the gastrointestinal glands (31), where they are thought to differentiate into gastrointestinal epithelial cells while in close contact with epithelial cells (28). A tandem schedule of stem cell replacement in the mesenchymal tissue as well as in the two distinct glandular tissues may give rise to endogenous fluctuations in gastric and colorectal cancer incidences. New stem cells fixed in the stomach and colon require the stabilization of stem cell phenotypes (10, 11). Because the stomach-specific genes are more highly expressed than the colon-specific genes (32), stomach-fixed stem cells require stronger stabilization than colon-fixed stem cells and may have a chance to produce a high number of cancer-prone stem cells. The epigenetic schedule of stem cell replacement appears to have more influence on gastric carcinogenesis than on colorectal carcinogenesis.

Two distinct long-term phases indicated that the increase of cancer incidence was rapid in the first long-term phase in men and in the second long-term phase in women (**Figure 1C**). We calculated the growth rate of age-specific cancer incidence to observe the actual trend of fluctuation cycles. The growth rate curves of the two cancer types tended to reach a high peak at early short-term fluctuation cycles in each long-term phase (**Figure 4**). The troughs of growth rate curves were consistent with the boundary of the two distinct long-term phases. The peak and trough of growth rate curves suggested that cancer-prone stem cells in the stomach and the colon exponentially increased in the early stage of the two long-term phases. Thus, an exponential increase of cancerization-prone stem cells occurred around the same period throughout the gastrointestinal glandular structures, even though putative 20-year-interval fluctuations estimated in the two cancer types were in the opposite direction. It is likely that the two distinct long-term phases reflect the replacement of mesenchymal stem cells that are recruited into the stomach and colon simultaneously.

Synchronous gastric and colorectal cancer diagnoses dropped within a limited age range of 68 to 73 years in the second long-term phase. Assuming the sequential replacement of mesenchymal-glandular stem cells, there are a low number of new stem cells at the first short-term fluctuation during the long-term phase, with barely an overlap between the distinct tissue environments that differentially promote cancer evolution. The growth rate curves for gastric and colorectal cancers were decoupled at an age range between 68 and 73 years. This period corresponded exactly to the drop in the incidence rate of synchronous cancer. Therefore, synchronous gastric and colorectal cancer appears to be reduced as a consequence of decoupled short-term fluctuations and a few new stem cells.

Fluctuation cycles were statistically significant at many intervals in addition to the long and short terms described in this study (data not shown). Non-linear regression models implied that many other fluctuations were included in the linear increase trend of age-specific cancer incidence. The actual trend of fluctuation cycles was found to be highly heterogeneous when comparing the growth rates of age-specific incidences among the old, middle, and recent KNCSP year subgroups (**Figure 4B**). However, the ages associated with the lowest colorectal cancer growth rate curves (66–67 years) remained constant. The growth rate curves of gastric cancer reached their lowest point between the age ranges of 64 and 71 years. These findings confirmed the two distinct long-term phases as nonrandom events that were reproduced commonly in the three KNCSP year subgroups.

The heterogeneity of periodic fluctuation cycles imposes limitations on predictive application of epigenetic schedule for cancer occurrence. There appear to be several reasons for heterogeneous cycles of cancer occurrence. First, a set of random genetic events triggers the stochastic occurrence of cancer, masking the periodic occurrence. Second, gastric cancer cells have been known to undergo histological shift from diffuse to intestinal types (33). The histologic shift implicates that gastric cancer cells develop at various stages of stem cell differentiation from new stem cells to gastric stem cells. This can produce heterogenous fluctuation curves in cancer incidence. Third, the growth pattern of gastric cancer lesion, especially infiltrating growth pattern, may lead to variations in detection of early cancer cells. Fourth, *H. pylori* eradication is likely to affect the periodic replacement of cancer-prone stem cells. Thus, it is challenging to analyze the age-related cancer incidence at short intervals depending on the histological type, the growth pattern, and the preventive measures of gastric cancer.

Taken together, large-scale data obtained from the KNCSP provided sufficient information about the periodic incidences of gastric and colorectal cancers. It has been long believed that stochastic accumulation of genetic alterations triggers an age-dependent exponential increase in cancer diagnoses. Accordingly, log-linear regression models for stochastic events were more accurate in both men and women with colorectal cancers. Age-specific incidences of gastrointestinal cancers periodically fluctuated at long-term (20 years) and short-term (8-year) intervals when using non-linear regression models. In particular, the non-stochastic model showed more accurate prediction performance in men with gastric cancer compared with the stochastic model. Actual incidence fluctuations demonstrated the coupling between gastric and colorectal cancers in the first long-term phase in men. Actual fluctuations were decoupled in the second long-term phase, during which the incidence of synchronous cancers dropped. These findings indicate that age-dependent increase of gastrointestinal cancer regularly fluctuated as a result of periodic replacement of mesenchymal and glandular stem cells.

## Data Availability Statement

The raw data supporting the conclusions of this article will be made available by the authors, without undue reservation.

## Ethics Statement

The studies involving human participants were reviewed and approved by The Institutional Review Board of the Songeui Campus, The Catholic University of Korea. Written informed consent for participation was not required for this study in accordance with the national legislation and the institutional requirements.

## Author Contributions

M-GR, J-HO, and S-JH conceptualized the article and drafted the manuscript. TK, J-SK, and S-JH performed data analysis. TK, J-SK, and YR guided the study and participated in discussions and preparation of the manuscript. All authors contributed to the article and approved the submitted version.

## Funding

This study was supported by Basic Science Research Program through the National Research Foundation of Korea funded by the Ministry of Education (2015R1D1A101059548) and Catholic Medical Center Research Foundation made in the program year of 2018 (5-2017-B0001-00268).

## Conflict of Interest

The authors declare that the research was conducted in the absence of any commercial or financial relationships that could be construed as a potential conflict of interest.
