# Supplementary material for: Periodic Fluctuations in the Incidence of Gastrointestinal Cancer
Source: Front Oncol. 2021 Mar 23;11:558040. doi: 10.3389/fonc.2021.558040 (PMC8021916; doi:10.3389/fonc.2021.558040)
Supplement: Supplementary file 1 [file DataSheet_1.docx]

**Table S1** Statistics of the invited recipients, participants, and new cancer patients during Korean National Cancer Screening Program for gastric cancer 2009–2016

| **Year** | **Sex** | **Number of invited recipients** | **Participants** | |  | **New gastric cancer patients** | |
| --- | --- | --- | --- | --- | --- | --- | --- |
|  |  |  | **Number** | **Rate (%)** |  | **Number** | **Incidence rate^1^** |
| 2009 | Men | 5,566,015 | 2,204,761 | 39.6 |  | 4,380 | 199 |
|  | Women | 5,545,373 | 2,822,429 | 50.9 |  | 2,154 | 76 |
|  | Total | 11,111,388 | 5,027,190 | 45.2 |  | 6,534 | 130 |
| 2010 | Men | 5,237,779 | 2,252,039 | 43.0 |  | 4,796 | 213 |
|  | Women | 5,061,238 | 2,819,333 | 55.7 |  | 2,329 | 83 |
|  | Total | 10,299,017 | 5,071,372 | 49.2 |  | 7,125 | 140 |
| 2011 | Men | 5,879,176 | 2,572,686 | 43.8 |  | 5,497 | 214 |
|  | Women | 6,346,819 | 3,283,833 | 51.7 |  | 2,771 | 84 |
|  | Total | 12,225,995 | 5,856,519 | 47.9 |  | 8,268 | 141 |
| 2012 | Men | 5,488,454 | 2,642,609 | 48.1 |  | 5,762 | 218 |
|  | Women | 5,657,908 | 3,292,927 | 58.2 |  | 2,689 | 82 |
|  | Total | 11,146,362 | 5,935,536 | 53.3 |  | 8,451 | 142 |
| 2013 | Men | 5,463,930 | 2,658,950 | 48.7 |  | 5,899 | 222 |
|  | Women | 5,847,102 | 3,358,080 | 57.4 |  | 2,975 | 89 |
|  | Total | 11,311,032 | 6,017,030 | 53.2 |  | 8,874 | 147 |
| 2014 | Men | 5,631,015 | 2,844,775 | 50.5 |  | 6,081 | 214 |
|  | Women | 5,927,325 | 3,512,634 | 59.3 |  | 2,892 | 82 |
|  | Total | 11,558,340 | 6,357,409 | 55.0 |  | 8,973 | 141 |
| 2015 | Men | 5,732,593 | 3,075,682 | 53.7 |  | 6,224 | 202 |
|  | Women | 6,122,281 | 3,703,154 | 60.5 |  | 3,040 | 82 |
|  | Total | 11,854,874 | 6,778,836 | 57.2 |  | 9,264 | 137 |
| 2016 | Men | 5,999,938 | 3,317,185 | 55.3 |  | 6,716 | 202 |
|  | Women | 6,330,760 | 3,943,221 | 62.3 |  | 3,232 | 82 |
|  | Total | 12,330,698 | 7,260,406 | 58.9 |  | 9,948 | 137 |
| Total | Men | 44,998,900 | 21,568,687 | 47.9 |  | 45,355 | 210 |
|  | Women | 46,838,806 | 26,735,611 | 57.1 |  | 22,082 | 83 |
|  | Total | 91,837,706 | 48,304,298 | 52.6 |  | 67,437 | 140 |

^1^Rate per 100,000 participants.

**Table S2** Statistics of the invited recipients, participants, and new cancer patients during Korean National Cancer Screening Program for colorectal cancer 2009–2016

| **Year** | **Sex** | **Number of invited recipients** | **Participants** | |  | **New colorectal cancer patients** | |
| --- | --- | --- | --- | --- | --- | --- | --- |
|  |  |  | **Number** | **Rate (%)** |  | **Number** | **Incidence rate^1^** |
| 2009 | Men | 3,392,163 | 1,019,863 | 30.1 |  | 931 | 91 |
|  | Women | 3,719,810 | 1,291,688 | 34.7 |  | 436 | 34 |
|  | Total | 7,111,973 | 2,311,551 | 32.5 |  | 1,367 | 59 |
| 2010 | Men | 3737668 | 1,291,842 | 34.6 |  | 1,156 | 89 |
|  | Women | 3909420 | 1,531,020 | 39.2 |  | 515 | 34 |
|  | Total | 7,647,088 | 2,822,862 | 36.9 |  | 1,671 | 59 |
| 2011 | Men | 4,045,914 | 1,391,123 | 34.4 |  | 1,203 | 86 |
|  | Women | 4,269,267 | 1,708,451 | 40.0 |  | 676 | 40 |
|  | Total | 8,315,181 | 3,099,574 | 37.3 |  | 1,879 | 61 |
| 2012 | Men | 6,450,978 | 1,786,530 | 27.7 |  | 1,406 | 79 |
|  | Women | 7,300,024 | 2,171,124 | 29.7 |  | 744 | 34 |
|  | Total | 13,751,002 | 3,957,654 | 28.8 |  | 2,150 | 54 |
| 2013 | Men | 6,263,384 | 1,956,586 | 31.2 |  | 1,640 | 84 |
|  | Women | 6,857,651 | 2,336,876 | 34.1 |  | 810 | 35 |
|  | Total | 13,121,035 | 4,293,462 | 32.7 |  | 2,450 | 57 |
| 2014 | Men | 6,423,733 | 2,111,301 | 32.9 |  | 1,926 | 91 |
|  | Women | 6,963,083 | 2,513,890 | 36.1 |  | 1,056 | 42 |
|  | Total | 13,386,816 | 4,625,191 | 34.6 |  | 2,982 | 64 |
| 2015 | Men | 6,732,080 | 2,364,591 | 35.1 |  | 1,962 | 83 |
|  | Women | 7,345,519 | 2,745,609 | 37.4 |  | 1,016 | 37 |
|  | Total | 14,077,599 | 5,110,200 | 36.3 |  | 2,978 | 58 |
| 2016 | Men | 6,992,355 | 2,537,560 | 36.3 |  | 1,978 | 78 |
|  | Women | 7,609,761 | 2,979,382 | 39.2 |  | 1,096 | 37 |
|  | Total | 14,602,116 | 5,516,942 | 37.8 |  | 3,074 | 56 |
| Total | Men | 44,038,275 | 14,459,396 | 32.8 |  | 12,202 | 84 |
|  | Women | 47,974,535 | 17,278,040 | 36.0 |  | 6,349 | 37 |
|  | Total | 92,012,810 | 31,737,436 | 34.5 |  | 18,551 | 58 |

^1^Rate per 100,000 participants.
